# Identification of intrinsic subtype-specific prognostic microRNAs in primary glioblastoma

**DOI:** 10.1186/1756-9966-33-9

**Published:** 2014-01-19

**Authors:** Rui Li, Kaiming Gao, Hui Luo, Xiefeng Wang, Yan Shi, Qingsheng Dong, WenKang Luan, Yongping You

**Affiliations:** 1 Department of Neurosurgery, The First Affiliated Hospital of Nanjing Medical University, No.300, Guangzhou Road, Gulou District, Nanjing 210029, PR China

**Keywords:** Glioblastoma, miRNA, Prognostic stratification

## Abstract

**Background:**

Glioblastoma multiforme (GBM) is the most malignant type of glioma. Integrated classification based on mRNA expression microarrays and whole–genome methylation subdivides GBM into five subtypes: Classical, Mesenchymal, Neural, Proneural-CpG island methylator phenotype (G-CIMP) and Proneural-non G-CIMP. Biomarkers that can be used to predict prognosis in each subtype have not been systematically investigated.

**Methods:**

In the present study, we used Cox regression and risk-score analysis to construct respective prognostic microRNA (miRNA) signatures in the five intrinsic subtypes of primary glioblastoma in The Cancer Genome Atlas (TCGA) dataset.

**Results:**

Patients who had high-risk scores had poor overall survival compared with patients who had low-risk scores. The prognostic miRNA signature for the Mesenchymal subtype (four risky miRNAs: miR-373, miR-296, miR-191, miR-602; one protective miRNA: miR-223) was further validated in an independent cohort containing 41 samples.

**Conclusion:**

We report novel diagnostic tools for deeper prognostic sub-stratification in GBM intrinsic subtypes based upon miRNA expression profiles and believe that such signature could lead to more individualized therapies to improve survival rates and provide a potential platform for future studies on gene treatment for GBM.

## Introduction

Glioblastoma multiforme (GBM) is the most lethal type of glioma in adults due to its poor prognosis and resistance to clinical therapy [1]. Despite the continuous improvements in radiotherapy, chemotherapy, and surgical treatments, the prognosis remains unsatisfactory [2]. Obstacles result, in part, from the heterogeneous nature and variable genetic aberrations among affected individuals. Developments of molecular biology offer a potential gene therapy for GBM. With the deepening of the research, a growing number of potential gene therapy targets have been identified, which calls for an urgent and objective classification based on molecular signatures rather than the histopathologic classification system [3]. Currently, many databases, such as The Cancer Genome Atlas (TCGA) Research Network, are being continuously improved and provide us with a comprehensive catalogue of genomic information [4]. Several molecular-based classification systems, such as mRNA expression-based and DNA methylation-based systems, have identified abnormalities driving tumorigenesis and correlated clinical data of samples.

The generally accepted molecular classification divides GBM into five subtypes: Proneural-G-CIMP, Proneural-non G-CIMP, Neural, Classical and Mesenchymal. Each subtype exhibits distinct biological behavior and characteristic gene expression [5,6]. However, biomarkers, which can be used to predict prognosis in each subtype, have not been systematically investigated. In our study, we identified significant miRNAs associated with clinical outcomes in each subtype. The prognostic miRNA signature for the Mesenchymal subtype was validated in an independent cohort containing 41 samples. The findings reveal the potential prognostic meaning of each subtype and provide an alternative individual treatment for patients with GBM.

## Methods

### Datasets

The miRNA expression microarray data (Level 3) for GBM samples were downloaded from The Cancer Genome Atlas (TCGA) database (http://cancergenome.nih.gov). The corresponding clinical data with the molecular subtype annotations were obtained from Ref [6]. In total, 448 primary GBM cases with molecular annotations (120 cases of Classical subtype, 141 cases of Mesenchymal subtype, 73 cases of Neural subtype, 29 cases Proneural-G-CIMP and 85 cases of Proneural-non G-CIMP) and miRNA expression microarray data were included in our analysis. A total of 41 Mesenchymal primary GBM from the Chinese Glioma Genome Atlas (CGGA) were used as a validation cohort by qRT-PCR assay.

### RNA isolation

Total RNA (tRNA) was extracted from frozen tissues using the mirVana miRNA Isolation Kit (Ambion, Inc., Austin, Tex), and its concentration and quality were determined with a NanoDrop ND-1000 spectrophotometer (NanoDrop Technologies, Wilmington, Del) [7].

### Real-time quantification of miRNAs by stem-loop RT-PCR

For the TaqMan-based real-time reverse transcription polymerase chain reaction (RT-PCR) assays, an ABI 7300 HT Sequence Detection system (Applied Biosystems, Foster City, CA) was used. All primers and probes of hsa-miR-373, hsa-miR-296, hsa-miR-191, hsa-miR-602 and hsa-miR-223, and RNU6B endogenous controls for TaqMan miRNA assays were purchased from Applied Biosystems. Real-time PCR was performed as described by Ref. [8]. The relative gene expression was calculated via a 2^−ΔΔCt^ method [9].

### Statistical analysis

Based on the generally accepted molecular classification of GBM (the classical, mesenchymal, neural, proneural-G-CIMP and proneural-non G-CIMP subtypes), we first analyzed the internal prognosis stratification of the five subtypes based on the MGMT promoter methylator phenotype. Kaplan-Meier survival analysis was used to estimate the survival distributions. The log-rank test was used to assess the statistical significance between the stratified survival groups using GraphPad Prism 6.0 statistical software. P < 0.05 was considered significant.

The expression level of each miRNA (n = 470) was assessed by Cox regression analysis using the BRB array tools package developed by Richard Simon and the BRB-ArrayTools Development Team [10]. Permutation Tests were performed with 10,000 permutations to select genes that were significantly associated with overall survival. For the Classical subtype, seven miRNAs with permutation P-values < 0.05 were selected as the candidate genes; for Proneural-G-CIMP and Proneural-non G-CIMP subtypes, three and ten miRNAs with permutation P-values <0.05 were selected as candidate genes. For Neural subtypes, eight miRNAs with permutation P-values < 0.015 were selected as candidate genes, and for Mesenchymal subtypes, five miRNAs with permutation P-values < 0.01 were selected as candidate genes. The significant miRNAs were divided into risky and protective types. Risky miRNAs were defined as miRNAs with a hazard ratio for death greater than 1. In contrast, protective miRNAs were defined based on a hazard ratio for death less than 1.

Using these significant miRNAs, a risk-score formula for predicting survival was developed based on a linear combination of the gene expression level (expr) weighted by the regression coefficient derived from the univariate Cox regression analysis (β) [11,12]. The risk score for each patient was calculated as follows:

$$\begin{array}{ll} \mathrm{Risk}\;\mathrm{score} & ={\mathrm{expr}}_{\mathrm{gene}1}*\beta_{\mathrm{gene}1}+{\mathrm{expr}}_{\mathrm{gene}2}*\beta_{\mathrm{gene}2}\\ & \;+{\mathrm{expr}}_{\mathrm{gene}3}*\beta_{\mathrm{gene}3}+\ldots {\mathrm{expr}}_{\mathrm{gene}\;n}\\ & \;*\beta_{\mathrm{gene}\;n} \end{array}$$

For each subtype, patients were divided into high-risk and low-risk groups according to the cutoff value (median risk score); patients in high-risk group are expected to have a poor outcome. The Kaplan-Meier method was used to estimate overall survival time for the two groups. Differences in survival times were analyzed using the two-sided log rank test. The significant miRNAs remained the same in validation set.

SPSS 13.0 for Windows (SPSS, Inc., Chicago, Ill) was used to conduct survival analyses. All tests were 2-tailed, and the significance level was set at P <0 .05.

## Results

### Identification prognostic microRNA signatures in the five molecular subtypes of primary glioblastoma

**Figure 1:**
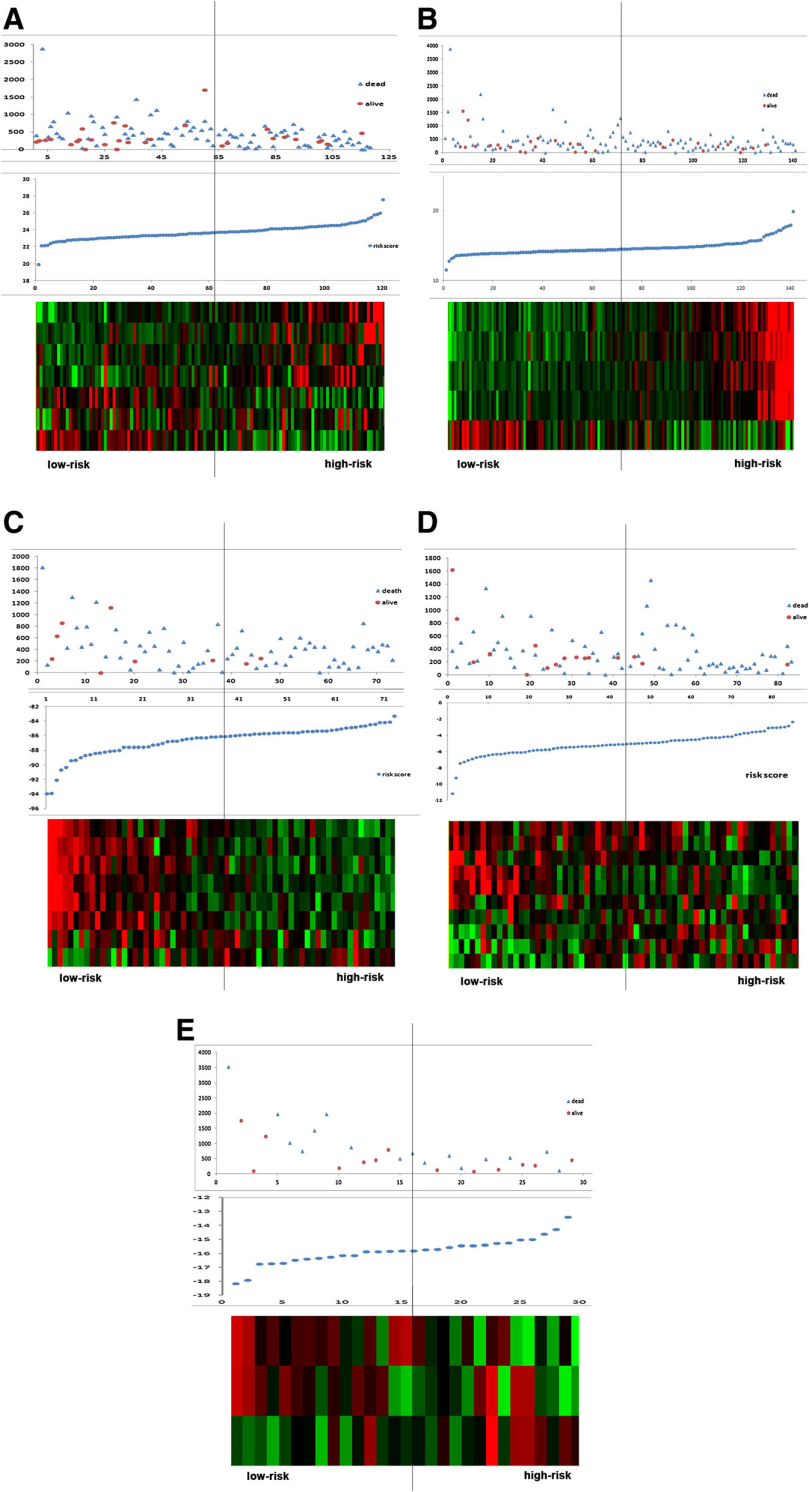
**Analysis of the microRNA (miRNA) signature risk score is illustrated for the five subtypes of GBM. (A)** Classical subtype; **(B)** Neural subtype; **(C)** Mesenchymal subtype; **(D)** Proneural-G-CIMP subtype; **(E)** Proneural-non G-CIMP subtype. (Top) Patient survival status and duration; (Middle) miRNA signature risk score distribution; (Bottom) heat map of ten miRNA expression profiles of patients with glioblastoma multiforme. The rows represent risky and protective miRNAs, and the columns represent patients. The vertical line represents the miRNA signature cutoff dividing patients into low-risk and high-risk groups.

**Figure 2:**
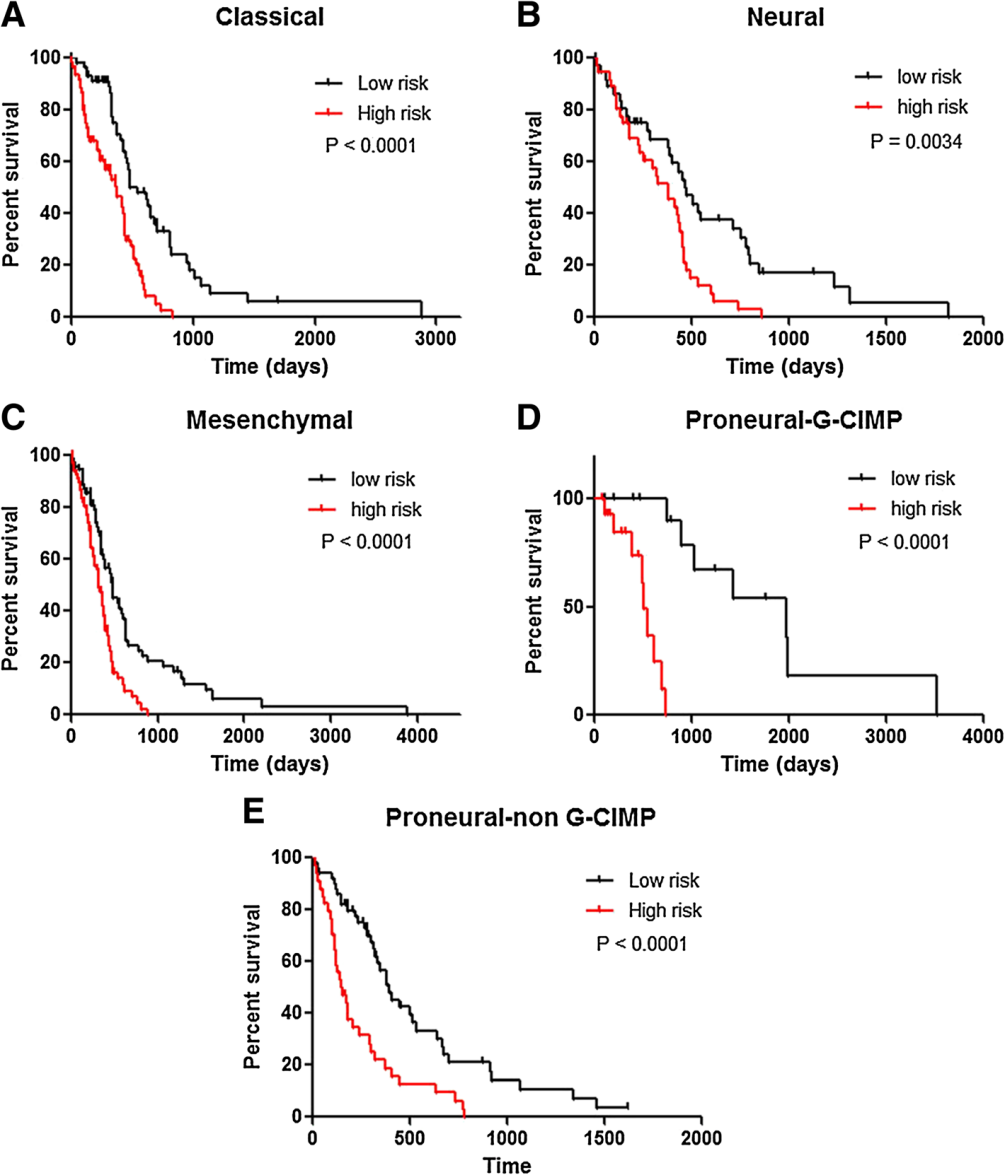
**Kaplan–Meier estimates of the overall survival of patients in each subtype of glioblastoma multiforme based on significant miRNAs. (A)** Classical subtype; **(B)** Neural subtype; **(C)** Mesenchymal subtype; **(D)** Proneural-G-CIMP subtype; **(E)** Proneural-non G-CIMP subtype. P-values < 0.05 was considered significant.

In classical subtype GBM, seven miRNAs (five risky miRNAs: hsa-miR-26a, hsa-miR-767-3p, hsa-miR-153, hsa-miR-31, hsa-miR-222, and two protective miRNAs: hsa-miR-654 and hsa-miR-422b) were found to be significantly correlated with clinical outcomes (p<0.05). The risk score was calculated based on the expression of these genes and was obtained in order to predict patient survival. A total of 120 samples were divided into a high-risk group (n = 60) and a low-risk group (n = 60) according to their risk score. The heatmap shows that, protective miRNAs exhibit high expression in low-risk group, while the risky miRNAs have high expression in high-risk group (Figure 1A). As shown in Figure 2A, the patients in the high-risk group suffered obvious worse overall survival than those in the low-risk group (P < 0.0001).

In another subtype of GBM, Neural, we filtered 8 miRNAs (one risky miRNAs: hsa-miR-222 and seven protective miRNAs: hsa-miR-422a, hsa-miR-662, hsa-miR-566, hsa-miR-24, hsa-miR-370, hsa-miR-492, hsa-miR-629) as signature genes for predicting the patient outcomes (p<0.015) (Figure 1B). Our result revealed that the prognoses of patients belonging to the high-risk group (n = 36) were worse than those of the low-risk group (n = 37) (Figure 2B).

A total of 5 miRNAs (four risky miRNAs: miR-373, miR-296, miR-191, miR-602; one protective miRNA: miR-223) were selected to identify the overall survival of patients in Mesenchymal subtype GBM (Figure 1C). Compared with the high-risk group, the low-risk group exhibited a better prognosis (p<0.001) (Figure 2C).

As for Proneural-G-CIMP and Proneural-non G-CIMP subtype GBM, three (one risky miRNAs: hsa-miR-582 and two protective miRNAs: hsa-miR-130a, hsa-miR-195) and ten miRNAs (four risky miRNAs: hsa-miR-335, hsa-miR-34a, hsa-miR-581, hsa-miR-21 and six protective miRNAs: hsa-miR-361, hsa-miR-145, hsa-miR-143, hsa-miR-378, hsa-miR-182, hsa-miR-183) were filtered for intrinsic prognostic analysis, respectively (Figure 1D-E). In both subtypes, the patients in the high-risk group exhibited shorter survival than patients in the low-risk group, as shown in Figure 2D-E.

### Validation of the prognostic value of the miRNA signatures of mesenchymal subtype in an independent cohort

**Figure 3:**
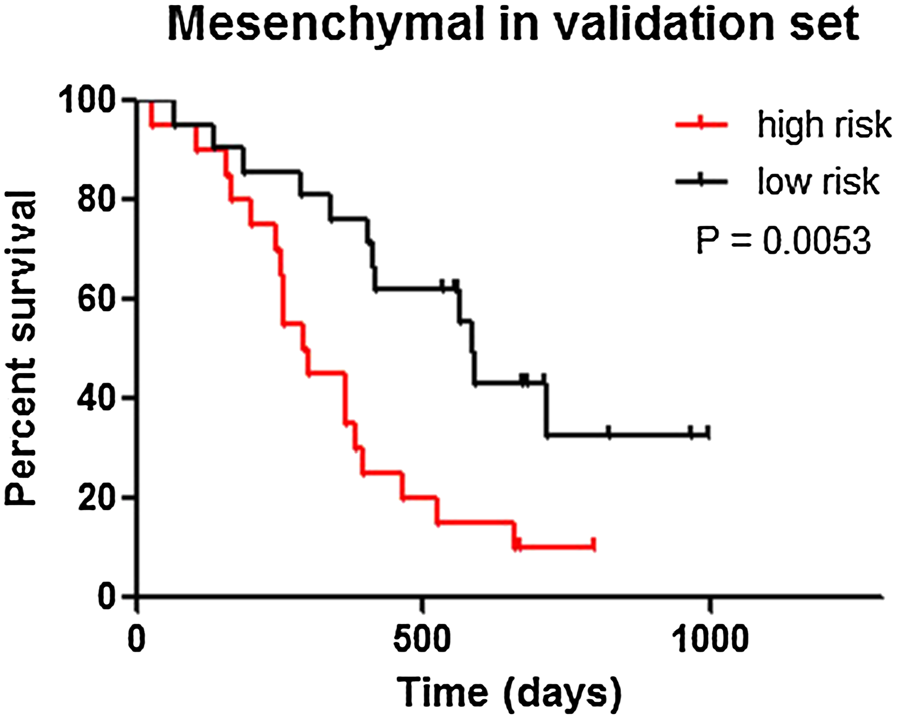
Validation of the prognostic value of gene signatures of the Mesenchymal subtype in an independent cohort by Kaplan–Meier analysis.

The prognostic miRNA signature for the Mesenchymal subtype was further validated in an independent cohort containing 41 Mesenchymal pGBM samples from CGGA. The expression levels of the five prognostic miRNAs (hsa-miR-373, hsa-miR-296, hsa-miR-191, hsa-miR-602 and hsa-miR-223) in the Mesenchymal subtype were analyzed by qRT-PCR in the independent cohort. The risk-score in each sample of validation cohort were calculated according the above formula. Furthermore, patients with Mesenchymal subtype GBM were divided into two groups based on their risk-scores. Similar results were obtained: the low-risk group exhibited extended survival, whereas the high-risk group exhibited shorter survival (Figure 3).

## Discussion

Glioblastoma multiforme (GBM) is the most malignant type of glioma. People who suffer from GBM exhibit poor prognosis. Survival ranges from one week to longer than three years, but most patients only survive approximately one year [13,14]. Decades of effort in clinical treatment have not satisfactorily improved prognosis. To improve the situation, researchers have begun to use molecular biological technologies to identify new approaches. Data on some important biomarkers in the progression of GBM, such as loss of heterozygosity (LOH) 10q, EGFR amplification, TP53 mutations, and PTEN mutations, have been collected and considered as certain gene targets [15,16]. To improve target efficiency, classifications of GBM have been established based on different gene signatures, including mRNA expression [17,18], microRNA expression, and methylation [19,20]. The generally accepted gene expression-based molecular classification by Phillips, H.S. et al. divides GBM into the Proneural, Neural, Classical and Mesenchymal subtypes [21]. Further studies divide the Proneural subtype of GBM into Proneural-G-CIMP and Proneural-non-G-CIMP subtypes based on the CPG Island methylator phenotype [22,23].

However, due to the complex mechanism of development and progression of GBM and the diversity of gene mutation, prognoses vary in samples of each subtype. The isocitrate dehydrogenase 1 (IDH1) mutation was reported to be a biomarker in the prediction of clinical outcomes for patients with GBM, and patients with IDH1 mutations exhibit better outcome than those with wild-type IDH1 in gliomas. However, the genomic alteration was frequent in grade II and grade III glial tumors but rare in primary glioblastoma (pGBM) [24,25]. Another prognostic biomarker, MGMT DNA methylation, was reported by Brennan CW et al. [6]. Only in classical subtype GBM, MGMT DNA methylation may be a predictive biomarker for treatment response. Patients with MGMT DNA methylation exhibit significantly better outcomes than atients with MGMT DNA unmethylation. However, the other four subtypes, Proneural-G-CIMP, Proneural-non G-CIMP, Neural, and Mesenchymal, exhibit no significant difference in clinical prognosis based on the MGMT DNA methylation biomarker. This prompted us to identify molecular prognostic markers that exist in all subtypes of GBM. In present study, we choose miRNAs as predictive biomarkers for intrinsic prognostic stratification. By using permutation tests and Cox regression analyses, significant genes were filtered and risk scores were calculated to help us divide patients into low- and high-risk groups. Our results revealed that in all five subtypes, patients belonging to the low-risk groups had significant longer overall survival than those in the high-risk group. Additionally, we validated the results in an independent database (CGGA) and obtained similar results. Although significant miRNAs were selected as predictive biomarkers for each subtype, there are many other factors, including age, sex, race and different treatment strategies, which may affect clinical outcomes and should also be taken into consideration in the treatment of glioblastoma.

In summary, our results indicate that miRNAs can be considered biomarkers for prognosis stratification in each subtype of glioblastoma, which could provide a novel approach in the evaluation of the prognosis and selection of the gene therapy targets.

## Competing interests

The authors declare that they have no competing interests.

## Authors’ contributions

LR, GKM, WXF performed the experiments. LH, SY, DQS, LWK analyzed the data. LR Wrote the manuscript. YYP, LR conceived and designed the experiments. All authors read and approved the final manuscript.
